# Editorial: Digital innovations in sustainable agri-food systems

**DOI:** 10.3389/fpls.2023.1304500

**Published:** 2023-11-07

**Authors:** Tiziana Amoriello, Chaojun Hou, Roberto Ciccoritti

**Affiliations:** ^1^ Research Centre for Food and Nutrition, Council for Agricultural Research and Economics, Rome, Italy; ^2^ School of Mathematics and Data Science, Zhongkai University of Agriculture and Engineering, GuangZhou, China; ^3^ Research Centre for Olive, Citrus and Tree Fruit, Council for Agricultural Research and Economics, Rome, Italy

**Keywords:** digital innovations, diseases and pest control, spectroscopy, quality monitoring, deep learning - artificial intelligence

Nowadays, agriculture and food production are increasingly being digitalized by using smart devices and intelligent software systems. This leads to an improvement in the economic, social, and environmental sustainability of agri-food value chains. In fact, digital technologies can offer powerful and efficient solutions to raise on-farm productivity, optimize environmental resource use, improve supply chain and logistics performances, reduce agri-food losses and waste, and increase competitiveness. This is possible by virtue of the availability of cost-effective, and highly interconnected hardware and software technologies, which collect, use, and analyse huge amounts of data. Consequently, information technology, decision support systems, the Internet of Things, robotics, computer vision and artificial intelligence applications play an essential role in modern business management. Predictive models, artificial intelligence and image analysis can be used to implement and develop innovative digital solutions for various applications in agri-food systems.

One of the most important applications regards the plant phenotyping, the disease management and yield assessment. For example, an efficient, rapid and accurate diagnosis method for apple foliar diseases is essential to promoting the development of the apple industry. However, these diseases are usually detected by visual inspection conducted by experts, which has high workloads, whereas an automated disease detection can improve accuracy and speed in detection. In this context, Xu and Wang applied image processing, data augmentation algorithms and a lightweight network model (ALAD-YOLO) to propose an accurate and lightweight model for apple leaf disease detection. Experimental results showed that the ALAD-YOLO architecture balanced detection speed and accuracy well, improving computational efficiency in comparison with existing models and meeting the requirements of real-time object detection for mobile devices.

A similar approach was adopted by Zhang W. et al. to develop an automatic crop pest control. In particular, the authors developed a refined YOLOv5 model (AgriPest-YOLO), based on deep learning, for monitoring agricultural pests with light traps, in order to achieve a balance between detection speed, accuracy and model size. This architecture improved the detection accuracy of the network for overlapping pests, extracting key features from pest images containing large background noise and dense distributions, and capturing detailed features for pest instances belonging to few samples and extreme small size.

Machine vision and the YOLO single-stage object detection network model were also used by Zhang Y. et al. to quickly detect wheat head with high-precision. These techniques were employed to develop an iOS-based intelligent wheat head counting mobile app, which could calculate the number of wheat heads in images shot in an agricultural environment in less than a second. The improvements made to the architecture, in terms of performance and accuracy, and the development of an easy-to-use app will allow it to be widely used in the agricultural production scenario.

To carry out an efficient and trusted prediction of wheat yield, Maji et al. developed a novel and automated two-staged methodology, called SlypNet, using advanced deep learning networks, which can extract various plant morphological features like spike and spikelet from the visual image of the wheat plant. By associating this information with spikelet density, it was possible to predict spikelet yield, as an estimate of grain yield, without using any auxiliary growth parameters.

High performance and automated solutions can be developed also to detect surface defects on agri-food products. Jiang et al. applied the artificial vision technology to the industrialization process of jujube production and the improvement in the level of automation. The authors proposed successfully a high-precision lightweight classification network, named JujubeNet, for the defect classification of jujubes. The implemented architecture can be extended to other defect classification scenarios.

The real-time assessment of food quality can be a crucial point for agri-food sector to reduce food waste and economic loss. Wen et al. presented a system based on computer vision to predict strawberry sugariness using non-destructive and affordable hardware. With respect to solutions of other studies, the authors highlighted the importance of temporal and environmental information, especially from up to fourteen days before the harvest, to ensure the model’s accuracy.

Finally, rapid, non-destructive, environment-friendly and high-throughput screening spectroscopy can be applied to control and evaluate food quality. Chen et al. reviewed all available literatures on the Infrared and Raman spectroscopy techniques to digitally detect the quality and safety of herbs across the whole process, from raw materials to patent herbal products. The presented information is very valuable for advance in automated monitoring of herbs and can contribute to the digital transformation of this industry.

## Author contributions

TA: Writing – original draft, Writing – review & editing. CH: Writing – review & editing. RC: Writing – review & editing.

